# Factors associated with vaccine hesitancy against COVID-19 among adults in Europe: a descriptive study analysis applying socio-ecological framework

**DOI:** 10.1186/s13104-024-06739-2

**Published:** 2024-03-19

**Authors:** Megumi Nagase

**Affiliations:** Friede-Springer-Endowed Professorship for Global Child Health, Witten/Herdecke University, Alfred-Herrhausen-Strasse 50, Witten, Germany

**Keywords:** Vaccine hesitancy, Europe, Socio-ecological model, COVID-19, SARS-CoV-2

## Abstract

**Objective:**

This study aimed to explore the factors associated with COVID-19 vaccine hesitancy in Europe among adults by using the Socio-Ecological Model.

**Results:**

This cross-sectional study used secondary data collected from respondents residing in 27 EU countries at the time of May 2021. The outcome was vaccine hesitancy against COVID-19, and the total sample size of 23,606 was analysed by binary logistic regression, as well as McKelvey and Zavonoia’s R^2^. After adding each level of variables, the model found the significant and increased association with vaccine hesitancy in younger age groups (21–39 years and 40–60 years vs. 65 years+), who left full-time education at a young age (16–19 years), those with manual jobs, those with children at home, individuals residing in small towns, and beliefs related to the vaccine. Together, the levels explained 49.5% of the variance associated with vaccine hesitancy, and the addition to each variable layer increased the variance. This highlights the need to consider broad factors at multiple levels to enhance vaccine acceptance and uptake.

## Introduction

In March 2020, soon after the declaration of the World Health Organization (WHO) declaration of the pandemic, Italy emerged as a major hot spot with the second-largest number of confirmed cases worldwide [1, 2], followed by dramatic increases in Spain and France [3]. By April 2020, Europe represented the largest share at 54.8% (621,407) of the total global confirmed cases, followed by the Americas at 27.8% (315,714) [4]. Responding to the alarming spread of COVID-19 across Europe, the WHO acknowledged that Europe had become the new epicentre of the pandemic, surpassing the rest of the world in reported cases and deaths combined [5].Despite the urgent need to prevent the further spread of COVID-19, European countries faced persistent vaccine hesitancy. WHO defines vaccine hesitancy as “a delay in acceptance or refusal of vaccines despite availability of vaccine services.” As of April 2021, 27% of European Union (EU) adult residents were vaccine-hesitant as they stated that they were either “very unlikely” or “rather unlikely” to receive the COVID-19 vaccine [6]. Given these considerations, it is imperative to examine further the attitude among people in Europe and the potential drivers of their unwillingness to be vaccinated against COVID-19. Thus, to better understand COVID-19 vaccine hesitancy, this study aims to explore the factors associated with individuals’ vaccine hesitancy against COVID-19 among adults in Europe.

This study applies the Socio-Ecological Model (SEM), which proposes that various factors at multiple levels shape individuals’ behaviour, ranging from people to groups to their socio-physical milieus [7]. In recent years, an increased number of studies [8–12] have leveraged the SEM model to identify elements associated with health behaviours, including vaccine uptake attitude. We adopted the four-level model [13], which includes more relevant pillars to the original. In the four-layer model, they start at the *individual* level and spread out to the *relationship*, *community*, and *societal* levels.

## Methods

### Data source and study design

This study primarily used cross-sectional survey data from Flash Eurobarometer 494: Attitudes on vaccination against COVID-19 [14]. IPSOS gathered data in each EU country between May 21 and May 26, 2021 and European Commission published the collected data in July 2021. The sampling was based on quotas of nationally representative samples. Interviews took place via surveys by country using IPSOS online panels and their partner networks. Except for Luxembourg, Cyprus, and Malta, each of the 27 EU countries included a sample size of approximately 1,000 citizens. To optimal utilization of the categorized education variable, which included the data of respondents who finished full-time education up to 15 and 16–19 years, we excluded the samples aged between 15 and 20. Additionally, the dataset combined external sources to expand the variety of societal variables based on the respondent´s country of origin. Those added variables encompass the country´s gross national income (GNI) per capita [15], the most commonly accepted religion [16], political spectrum [17], and geographical region of the country [18]. After the data cleaning, we set the final sample size to 23,606.

### Outcome variables

The outcome variable of interest was COVID-19 vaccine hesitancy. To determine whether the respondent is vaccine-hesitant or not, this study used the aforementioned WHO definition of vaccine hesitancy. Based on that, responses to the question “When would you like to get vaccinated?” were categorized as follows: those who answered with “sometime in 2021,” “later,” “don’t know,” and “never” were classified as vaccine-hesitant group, while responses such as “I have already been vaccinated” and “as soon as possible” were placed in the vaccine-acceptant group.

### Exposure variables

Exposure variables consist of individual, relationship, community and societal levels. The individual category included each respondent’s gender, age, age when they stopped full-time education and occupational status. Additionally, we incorporated the respondents´ beliefs about benefits and safety, efficacy, and subjective norm of COVID-19 vaccines as variables placed at the individual level. These belief variables include questions exploring whether they agree or disagree on “Benefits of COVID-19 vaccines outweigh the possible risks,” “You can avoid being infected by COVID-19 without being vaccinated,” or “Everyone should get vaccinated against COVID-19, it is a civic duty. " We identified the number of people aged 15 and older and children younger than 15 in the household as relationship variables, that represent household influence. The community-level variable examined respondents’ type of residence, reflecting their subjective urbanization. Finally, the societal level considered their country’s cultural, social and political characteristics as mentioned in the section of data source above.

### Statistical analysis

We used Stata version 17 for data analysis and conducted unweighted univariate analysis, Pearson’s chi-square (Table 1), and Multivariate binary logistic regression (Table 2) to assess the association between the outcome and explanatory variables. For binary logistic regression, we examined different levels of variables in a stepwise process to observe the significance and strength of each level of variables and their impact associated with the outcome. Finally, the analysis reported the value of McKelvey-Zavoina pseudo-R^2^ [19] at each level of the logistic regression model. While in logistic regression, pseudo-R^2^ does not estimate the variance explained by variables as it does in ordinary least squares (OLS) regression, the McKelvey-Zavoina R^2^ provides a value that can be interpreted in a way similar to the OLS regression R^2^ [11, 20].

**Table 1:** Bivariate analysis results, flash eurobarometer 494: attitudes on vaccination against COVID-19 (July 2021), *N* = 22,078

			Vaccine Hesitancy				
			Yes		No		
*Individual level*	N (%)		n (%)		n (%)		
Total	22,078	(100)	7,125	(32.27)	14,953	(67.73)	
**Gender** ^**1**^							< 0.001
Male	10,857	(49.28)	3,292	(30.32)	7,565	(69.68)	
Female	11,175	(50.72)	3,807	(34.07)	7,368	(65.93)	
**Age** ^**2**^							< 0.001
21–39	7,609	(34.46)	3,515	(46.20)	4,094	(53.80)	
40–64	10,176	(46.09)	3,021	(29.69)	7,155	(70.31)	
65 and over	4,293	(19.44)	589	(13.72)	3,704	(86.28)	
**Age when stopped** **full-time education** ^**3**^							< 0.001
Up to 15 years	703	(3.46)	199	(28.31)	504	(71.70)	
16–19	6,928	(34.06)	2,315	(33.42)	4,613	(66.58)	
20 years	11,031	(54.23)	3,325	(30.14)	7,706	(69.86)	
Still studying	1,679	(8.25)	614	(36.57)	1,065	(63.43)	
**Occupation** ^**4**^							< 0.001
Self-employed	2,503	(11.82)	950	(37.95)	1,553	(62.05)	
Employee	10,985	(51.87)	3,653	(33.25)	7,332	(66.75)	
Manual worker	1,193	(5.63)	596	(49.96)	597	(50.04)	
Unemployed	6,496	(30.67)	1,649	(25.38)	4,847	(74.62)	
*Individual- Beliefs*							
**Benefits/Safety** ^**5**^							< 0.001
Agree	16,902	(80.80)	3,284	(19.43)	13,618	(80.57)	
Disagree	4,016	(19.20)	3,168	(78.84)	848	(21.12)	
**Efficacy** ^**6**^							< 0.001
Agree	10,090	(50.14)	1,683	(16.68)	8,407	(83.32)	
Disagree	10,032	(49.86)	4,851	(48.36)	5,181	(51.64)	
**Subjective norm** ^**7**^							< 0.001
Agree	13,439	(63.54)	1,832	(13.63)	11,607	(86.37)	
Disagree	7,713	(34.46)	4,977	(64.53)	2,736	(35.47)	
*Relationship Level*							
**# of adults aged ≥ 15 in a household** ^**8**^							< 0.001
1	5,261	(25.18)	1,605	(30.51)	3,656	(64.49)	
2	9,507	(45.50)	2,807	(29.53)	6,700	(70.47)	
3+	6,127	(29.32)	2,149	(35.07)	3,978	(64.93)	
**# of children aged < 15 in a household** ^**9**^							< 0.001
0	14,942	(71.20)	4,123	(27.59)	10,819	(72.41)	
1	3,529	(16.82)	1,432	(40.58)	2,097	(59.42)	
2+	2,515	(11.98)	1,050	(41.75)	1,465	(58.25)	
*Community Level*							
**Subjective urbanization** ^**10**^							0.017
Rural area	5,413	(24.52)	1,759	(32.50)	3,654	(67.50)	
Small or medium- sized town	8,810	(39.90)	2,922	(33.17)	5,888	(66.83)	
Large town/city	7,855	(35.58)	2,444	(31.11)	5,411	(68.89)	
*Societal Level*							
**GNI per capita** ^**11**^							< 0.001
Above EU average	8,097	(36.67)	1,926	(23.79)	6,171	(76.21)	
Below EU average	13,981	(63.33)	5,199	(37.19)	8,782	(62.81)	
**Religion** ^**12**^							< 0.001
Catholic	12,957	(58.69)	3,974	(30.67)	8,983	(69.33)	
Protestant	3,424	(15.51)	1,059	(30.93)	2,365	(69.07)	
Orthodox	2,181	(9.88)	1,077	(49.38)	1,104	(50.62)	
Unspecified or none	3,516	(15.93)	1,015	(28.87)	2,501	(71.13)	
**Political ideology** ^**13**^							< 0.001
Center left	4,429	(20.06)	1,513	(34.16)	2,916	(65.84)	
Center right	4,558	(20.64)	1,517	(33.28)	3,041	(66.72)	
Right	13,091	(59.29)	4,095	(31.28)	8,996	(68.72)	
**Geographical region** ^**14**^							
Western Europe	12,645	(52.27)	3,094	(24.47)	9,551	(75.53)	< 0.001
Eastern Europe	9,433	(42.73)	4,031	(42.73)	5,402	(57.27)	

^1^*N*=22,032 (missing data = 46), ^2^*N*=22,078, ^3^*N*=20,341 (missing data = 1,737), ^4^*N*= 21,177 (missing data = 901), ^5^*N*=20,918 (missing data = 1,160), ^6^*N*=20,122 (missing data = 1,956), ^7^*N*=21,152 (missing data = 926), ^8^*N*=20,895 (missing data = 1,183), ^9^*N*=20,986 (missing data = 1,092), ^10–14^*N*=22,078

**Table 2:** Multivariate binary logistic regression analysis results and McKelvey and Zavoina’s R^2^, Flash Eurobarometer 494: Attitudes on vaccination against COVID-19 (July 2021)

	M1 (95% C.I.)	M2 (95% C.I.)	M3 (95% C.I.)	M4 (95% C.I.)	M5 (95% C.I.)	Unadjusted OR (95% C.I.)
*Individual* (pseudo-R^2^ = 0.108)						
**Gender**						
Male	(ref)	(ref)	(ref)	(ref)	(ref)	(ref)
Female	1.16** (1.09–1.24)	1.05 (0.96–1.14)	1.07 (0.98–1.16)	1.07 (0.98–1.17)	1.08 (0.99–1.18)	1.19** (1.12–1.26)
**Age (years)**						
65+	(ref)	(ref)	(ref)	(ref)	(ref)	(ref)
40–64	2.77** (2.46–3.11)	2.24** (1.91–2.62)	2.10** (1.78–2.47)	2.10** (1.79–2.47)	2.09** (1.77–2.47)	2.66** (2.41–2.93)
21–39	5.73** (5.07–6.46)	4.12** (3.51–4.85)	3.76** (3.16–4.46)	3.76** (3.17–4.46)	3.75** (1.77–2.47)	5.40** (4.90–5.95)
**Age when stopped full-time education**						
20+	(ref)	(ref)	(ref)	(ref)	(ref)	(ref)
16–19	1.27** (1.18–1.36)	1.16** (1.05–1.27)	1.16** (1.05–1.27)	1.16* (1.05–1.28)	1.19** (1.08–1.32)	1.16** (1.09–1.24)
Up to 15	1.16 (0.97–1.40)	1.17 (0.91–1.51)	1.10 (0.84–1.44)	1.11 (0.85–1.45)	1.33 (1.01–1.74)	0.92 (0.77–1.08)
Still studying	0.91 (0.81–1.02)	0.98 (0.84–1.14)	0.93 (0.79–1.10)	0.93 (0.79–1.10)	0.98 (0.83–1.16)	1.34** (1.20–1.49)
**Occupation**						
Unemployed	(ref)	(ref)	(ref)	(ref)	(ref)	(ref)
Employee	0.90* (0.83–0.98)	0.87* (0.77-0-97)	0.83** (0.74-0-94)	0.83** (0.73–0.93)	0.83** (0.74–0.94)	1.46** (1.37–1.57)
Self-employed	1.28** (1.14–1.43)	1.15 (0.99–1.33)	1.13 (0.97–1.32)	1.13 (0.97–1.32)	1.05 (0.89–1.23)	1.80** (1.63–1.98)
Manual worker	1.66** (1.43–1.91)	1.53** (1.26–1.86)	1.43** (1.17–1.76)	1.43** (1.16–1.75)	1.26* (1.02–1.55)	2.93** (2.59–3.33)
*Beliefs* (pseudo-R^2^ = 0.423)						
**Benefits/safety**						
Agree		(ref)	(ref)	(ref)	(ref)	(ref)
Disagree		6.11** (5.48–6.81)	6.38** (5.70–7.15)	6.40** (5.71–7.17)	6.33** (5.64–7.11)	15.49** (14.23–16.86)
**Efficacy**						
Agree		(ref)	(ref)	(ref)	(ref)	(ref)
Disagree		2.38** (2.81–2.60)	2.33** (2.13–2.56)	2.33** (2.12–2.55)	2.34** (2.13–2.57)	4.68** (4.38–4.99)
**Subjective norm**						
Agree		(ref)	(ref)	(ref)	(ref)	(ref)
Disagree		4.99** (4.57–5.46)	5.00** (4.56–5.49)	5.01** (4.57–5.50)	4.70** (4.27–5.18)	11.53** (10.77–12.33)
*Relationship* (R^2^ = 0.026)						
**# of adults in a household**						
1			(ref)	(ref)	(ref)	(ref)
2			1.00 (0.89–1.11)	1.00 (0.90–1.12)	0.98 (0.87–1.10)	0.95 (0.89–1.03)
3+			1.15* (1.02–1.30)	1.16* (1.03–1.30)	1.04 (0.92–1.17)	1.23** (1.14–1.33)
	M1	M2	M3	M4	M5	Unadjusted OR
**# of children in a household**						
0			(ref)	(ref)	(ref)	(ref)
1			1.28** (1.16–1.42)	1.28** (1.16–1.42)	1.22** (1.11–1.35)	1.81 ** (1.70–1.93)
2+			1.55** (1.20–2.01)	1.56** (1.21–2.03)	1.65** (1.27–2.14)	2.05** (1.72–2.45)
*Community* (pseudo-R^2^ = 0.001)						
**Residence**						
Large city				(ref)	(ref)	(ref)
Small town				1.03 (0.93–1.14)	1.16** (1.05–1.29)	1.10* (1.03–1.17)
Rural area				0.91 (0.81–1.029	1.12 (1.00-1.27)	1.07 (0.99–1.15)
*Societal* (pseudo-R^2^ = 0.074)						
**GNI**						
Above EU average					(ref)	(ref)
Below EU average					1.23** (1.05–1.43)	1.90** (1.78 − 0.02)
**Religion**						
Unspecified					(ref)	(ref)
Protestant					1.44** (1.21–1.70)	1.10 (1.00-1.22)
Catholic					1.10 (0.97–1.26)	1.09* (1.00-1.18)
Orthodox					2.69** (2.22–3.29)	2.40** (2.15–2.69)
**Political spectrum**						
Right					(ref)	(ref)
Centre-right					0.91 (0.80–1.04)	1.10 (1.02–1.18)
Centre-left					1.02 (0.90–1.16)	1.14** (1.06–1.23)
**Region**						
Western Europe					(ref)	(ref)
Eastern Europe					1.55** (1.36–1.77)	2.30** (2.17–2.44)
Accumulated R2	-	0.465	0.470	0.471	0.495	-
AIC	1.179	0.820	0.806	0.806	0.789	-
N Observations	19,583	16,899	16,128	16,128	16,128	-

* p-value < 0.05, ** p-value < 0.01

95% C.I. = 95% confidence interval

## Results

### Bivariate associations with COVID-19 vaccine hesitancy

Results from chi-square testing (Table 1), stratified by vaccine uptake attitude, revealed that all the listed variables were associated with the outcomes, with a p-value of less than 0.01, except the community level. In the vaccine-hesitant group, there was a slightly higher proportion of females (34.0%), and the 21–39 age group displayed the highest rate of vaccine hesitancy (46.2%). In contrast, 86.2% of respondents in the oldest age group (65 years+) expressed willingness to be vaccinated. Among the belief variables, individuals who doubted the benefits and safety of the vaccine exhibited the highest proportion of the outcome at 78.8%, followed by disagreement with the subjective norm of getting vaccinated (64.5%). Conversely, responses indicating agreement or disagreement with the vaccine’s efficacy were nearly equal, resulting in the lowest proportion of vaccine hesitancy at 48.3% within the belief variables.

A smaller proportion of households without children under 15 years showed vaccine hesitancy at 27.6%, but this changed when people lived with one child (40.6%) or more than two children (41.8%), as they became more hesitant to the vaccine. Concerning societal factors, vaccine hesitancy was higher in the lower-GNI group (37.2%) than in the higher-GNI group (23.8%). The different religious groups from Orthodox-dominant countries showed a particularly high tendency to be vaccine hesitant, amounting to almost half of the group. In addition, the proportion of vaccine hesitancy was almost twice as common among respondents from the Eastern region (42.7%) compared to those from the Western region (24.5%).

### Stepwise binary logistic regression modelling of COVID-19 vaccine hesitance

Table 2 provides the findings from the binary logistic regression analyses along with McKelvey and Zavoina’s R^2^. The adjusted odds ratio (OR) showed that the following factors consistently linked to a higher chance of vaccine hesitancy when compared to the reference group: (I) younger age group, (II) those who stopped full-time education between ages 16 and 19, (II) people involved in manual work, (IV) individuals who disagreed with the benefits, safety, and norms associated with vaccination, (V) households with more than one child, (VI) individuals residing in small towns. Within the age category, we observed the higher OR with a younger age group (OR = 3.75 in 21–39 years vs. OR = 2.09 in 40–64 years). Similarly, an increase in the number of children in the household was associated with higher odds of outcome (OR = 1.22 in one child household vs. OR = 1.65 in more than two children household). At the societal level, vaccine hesitancy was more likely in areas characterized by lower GNI, Protestant or Orthodox groups, and residing in Eastern Europe. Conversely, only employee status in the occupation variables showed a consistently strong negative association with the outcome. Individuals who finished full-time study for up to 15 years and are currently pursuing education, two-adult households, residence in rural areas and political spectrum at the societal level showed no statistical significance in any analytical models.

The belief variables had the greatest R^2^ (R^2^ = 0.423), followed by other individual demographic variables (R^2^ = 0.108), societal-level variables (R^2^ = 0.074), and relationship-level variables (R^2^ = 0.026). The smallest effect was observed at the community level (R^2^ = 0.001). We observed the smallest effect at the community level (R2 = 0.001). Further, starting at the individual level and adding to each layer of variables increased the variance explained in relation to the outcome from 0.108 to 0.495.

## Discussion

Applying the SEM, this study examined the factors associated with COVID-19 vaccine hesitancy among adults in EU countries. The key findings demonstrated that younger age, residence in small towns, completion of full-time education between the ages of 16 and 19, manual worker status, positive beliefs related to vaccination, and having more children in the household, demonstrated significant linkage to COVID-19 vaccine hesitancy. Only employee status had a negative association with the outcome, implicating that they had a lower chance to exhibit vaccine hesitancy compared to the reference group.

The results of the association between the outcome and younger age, as well residing in small towns, align with existing literature on determinants of vaccine hesitancy in single countries (e.g., Germany [8], Austria [21], France [22], Croatia [23]). In contrast, while education is widely acknowledged as one of the powerful determinants of health [24–27], this study shows a partial absence or entire absence of consistent association with the outcome. This discrepancy might be because the variable does not necessarily reflect the respondents’ educational level or schooling years, as other studies often do.

The results also highlight that the perceived benefits, safety, efficacy, and norms of getting vaccinated against COVID-19 had the most potent effect on an individual’s vaccine uptake attitudes. This emphasises the importance of psychological factors and need for effective health communication to combat misinformation in addressing widespread vaccine hesitancy. In addition, the findings demonstrate that not only belief factors and individual-level variables but also relationship-, community-, and societal-level of variables had either entire or partial significance. However, the values of R^2^ illustrate the varying effects of each level, with beliefs having the most substantial effect, followed by individual demographical variables, then the societal, relationship, and community levels. The varying effects at each level would be contingent on the available data and the methodology employed in selecting and categorizing the data into each level.

Finally, it is important to note that attitudes toward vaccine uptake can vary significantly based on specific contexts [28]. While our study identifies key factors related to the outcome, by using aggregated data from 27 EU countries, it´s highly probable that results will differ when conducting the same analysis for a single country. For example, a study already found that in Bulgaria, age was not a significant factor (OR = 0.98) in vaccine hesitancy, whereas in Sweden, it demonstrated a strong association (OR = 1.67) with a p-value less than 0.001 [29]. This underscores the importance of careful consideration for various contextual factors within each unit, whether it is a neighbourhood, municipality, state, or country.

## Conclusion

The reluctance of a considerable number of individuals to be vaccinated can significantly disrupt population efforts to achieve herd immunity [30, 31], increasing the risk of the next outbreak in future [32]. When developing effective measures to enhance vaccine acceptance and uptake, the results of this study suggest that factors at various levels surrounding individuals determine vaccine hesitancy. Therefore, accounting for the factors across multiple levels is crucial to boost vaccine acceptance and uptake.

### Limitations

This study identifies five major limitations. First, due to its nature as a cross-sectional study, we cannot determine any causal relationships between the outcome and the explanatory variables. Second, the self-reported survey introduces potential validity issues. Third, Global efforts to promote vaccination may lead to social desirability bias, affecting honest responses. Fourth, we performed only unweighted analysis and did not account for clustering issues when gathering the respondents from 27 different countries into one large sample. Fifth, no confounders were considered. Due to those limitations, the results are not be generalisable.

## Data Availability

The datasets generated and/or analysed during the current study are available in the GESIS repository, https://search.gesis.org/research_data/ZA7771.

## Abbreviations

WHO: World Health Organization
SEM: Socio-Ecological Model
GNI: Gross National Income
OLS: Ordinary Least Squares
OR: Odds ratio
MN: Megumi Nagase
